# Corrected whole blood biomarkers – the equation of Dill and Costill revisited

**DOI:** 10.14814/phy2.13749

**Published:** 2018-06-25

**Authors:** Pekka Matomäki, Heikki Kainulainen, Heikki Kyröläinen

**Affiliations:** ^1^ Faculty of Sport and Health Sciences Biology of Physical Activity University of Jyväskylä Jyväskylä Finland

**Keywords:** Biomarker, correction formula, Dill and Costill equation, plasma change

## Abstract

An exercise bout or a dehydration often causes a reduction in plasma volume, which should be acknowledged when considering the change in biomarkers before and after the plasma changing event. The classic equation from Dill and Costill (1974, J. Appl. Physiol., 37, 247–248) for plasma volume shift is usually utilized in such a case. Although this works well with plasma and serum biomarkers, we argue in this note that this traditional approach gives misleading results in the context of whole blood biomarkers, such as lactate, white cells, and thrombocytes. In this study, we demonstrate that to calculate the change in the total amount of circulating whole blood biomarker, one should utilize a formula

$$\frac{{\text{BM}}_{\mathrm{post}}}{{\text{BM}}_{\mathrm{pre}}}\times \frac{Hb_{\mathrm{pre}}}{Hb_{\mathrm{post}}}-1.$$

Here Hb and BM are, respectively, the concentrations for the hemoglobin and for the inspected whole blood biomarker before (pre) and after (post) the plasma changing incident.

## Abstract 2

In this study, a correction formula, induced by a plasma change, is derived for the whole blood biomarkers. Usually utilized correction formula from Dill & Costill is applicable only for the plasma and serum biomarkers. The difference between the two formulas is discussed.

## Introduction

It is a quite customary observation that during dehydration (Costill and Fink 1974; Nose et al. 1988) and exercise, both in endurance (Kingwell et al. 1997; Li and Gleeson 2004) as well as strength training (Collins et al. 1986; Kraemer et al. 1990), plasma volume decreases acutely causing the well‐known hemoconcentration effect (Harrison 1985). This flux of water from bloodstream is mainly induced by the increased osmotic pressure between blood vessels and extravascular space, as well as increased hydrostatic pressure in capillaries (Sjøgaard and Saltin 1982; Harrison 1985). If refueling is carried out, plasma volume is usually returned to a resting level within hours of recovery (Collins et al. 1986; Kingwell et al. 1997), although a plasma volume expansion is also a possible outcome (Astrand and Saltin 1964; Robach et al. 2014).

This acute loss in plasma volume causes increase in the concentration of blood biomarkers regardless of the possible responses from exercise. Hence, this phenomenon is important to acknowledge when comparing biomarkers before and after an exercise bout. This is usually done by applying an equation of relative plasma volume change given by Dill and Costill (1974). Although not strongly stressed, this approach is only suitable for plasma and serum biomarkers, whereas whole blood biomarkers would need an approach of their own. In this short note, we first give a brief summary of how the equation from (Dill and Costill 1974) is usually utilized when calculating corrected biomarker value. This is followed by our main contribution, which is the equation (eq. 5) for calculating the corrected change for the whole blood biomarkers. Our argument is accompanied by an illustrative example and a brief explanation why the traditional approach is not adequate.

### Calculating the change in plasma volume

Dill and Costill (1974) have provided the notorious method to calculate the change in plasma volume (PV). For the derivation, one needs hemoglobin concentration (Hb), hematocrit (Hct), and total blood volume (BV) before (pre) and after (post) the exercise bout. The celebrated equation from (Dill and Costill 1974) for the change in the plasma volume (ΔPV) is

(1) $$\Delta \text{PV}=\frac{{\text{PV}}_{\mathrm{post}}-{\text{PV}}_{\mathrm{pre}}}{{\text{PV}}_{\mathrm{pre}}}=\frac{Hb_{\mathrm{pre}}\times \left(1-\mathrm{Hct}{}_{\mathrm{post}}\right)}{Hb_{\mathrm{post}}\times \left(1-\mathrm{Hct}{}_{\mathrm{pre}}\right)}-1.$$

It is basically based on the assumption that the absolute mass of circulating red cells in bloodstream stays unchanged, that is,

(2) $$\begin{array}{cc} {\text{BV}}_{\mathrm{pre}} & \times Hb_{\mathrm{pre}}={\text{BV}}_{\mathrm{post}}\times Hb_{\mathrm{post}}\}\\ & \Rightarrow \frac{{\text{BV}}_{\mathrm{post}}}{{\text{BV}}_{\mathrm{pre}}}=\frac{Hb_{\mathrm{pre}}}{Hb_{\mathrm{post}}}. \end{array}$$

In practice, this is occasionally violated, for example, because of a footstrike‐induced hemolysis during a running exercise (Telford et al. 2003; Robach et al. 2014). The equation also implicitly assumes uniform vascular mixing which is sometimes compromised, for example, in clinical conditions such as chronic kidney disease (Lobigs et al. 2017).

## Results and Discussion

### Corrected value for plasma and serum biomarker

Most biomarkers are measured from plasma or serum, such as branched chain amino acids. For these biomarkers, reported usually as an amount in liter of a plasma or serum, customary way to make the plasma volume change correction is, for example, from (Alis et al. 2015),

(3) $${\text{PM}}_{\mathrm{post},c}={\text{PM}}_{\mathrm{post},u}\times \left(1+\Delta \text{PV}\right),$$

where PM_post,*c*_ and PM_post,*u*_ indicate corrected and uncorrected serum or plasma biomarker after the exercise, respectively. Another class of biomarkers from bloodstream are the whole blood biomarkers (BM). These are reported usually as an amount in the liter of a whole blood and they include, for example, lactate, white cells, thrombocytes, manganese, protein c, troponin T, and *α*‐glucosidase. In the same spirit to above, the right correction for these would be

(4) $${\text{BM}}_{\mathrm{post},c}={\text{BM}}_{\mathrm{post},u}\times \left(1+\Delta \text{BV}\right),$$

where ΔBV is the change in a total blood volume. However, ΔBV can be usually calculated only in clinical practice (D'Angelo et al. 2015; Lobigs et al. 2017), whence the practicality of equation (eq. 4) is quite limited. Moreover, the usage of plasma correction formula (eq. 3) in the case of whole blood biomarkers would give misleading results as it assumes, implicitly, that measured biomarker is reported as an amount in plasma or serum, and ΔPV differs from ΔBV.

### Corrected whole blood marker

To make a correction formula for whole blood biomarkers, define TBM to be the total amount of inspected whole blood biomarker. When BM is the amount of biomarker with respect to blood volume (e.g., lactate as mmol/L), TBM is the total amount of it in the whole bloodstream (e.g., total amount of lactate in the circulation as mmol). While corrected BM value cannot be determined with ease, the relative change of the total amount of the circulating whole blood biomarker (ΔTBM), which is often of interest, can nevertheless be calculated quite effortlessly. To calculate its change, it is first observed that the total amount of circulating blood biomarker TBM = BM × BV. Although this cannot be directly measured in any simple method, its relative change

(5) $$\begin{array}{cc} \Delta \text{TBM} & =\frac{{\text{TBM}}_{\mathrm{post}}-{\text{TBM}}_{\mathrm{pre}}}{{\text{TBM}}_{\mathrm{pre}}}\\ & =\frac{{\text{BM}}_{\mathrm{post}}\times {\text{BV}}_{\mathrm{post}}-{\text{BM}}_{\mathrm{pre}}\times {\text{BV}}_{\mathrm{pre}}}{{\text{BM}}_{\mathrm{pre}}\times {\text{BV}}_{\mathrm{pre}}}\\ & =\frac{{\text{BM}}_{\mathrm{post}}}{{\text{BM}}_{\mathrm{pre}}}\times \frac{{\text{BV}}_{\mathrm{post}}}{{\text{BV}}_{\mathrm{pre}}}-1=\frac{{\text{BM}}_{\mathrm{post}}}{{\text{BM}}_{\mathrm{pre}}}\times \frac{Hb_{\mathrm{pre}}}{Hb_{\mathrm{post}}}-1 \end{array}$$

is quite simple to reach, where the last equation follows from equation (eq 2). In fact, only a knowledge on Hb is needed for the relative change of the whole blood marker.

### The insufficiency of the traditional approach

The next example illustrates how the equation (eq. 5) can be used and how the use of plasma correction equation (eq. 3) can give misleading values with whole blood biomarkers indicating that the newly derived equation (eq. 5) should be preferred.

### Example

Assume a hypothetical case, where one is interested in a change of white cells before and after an exercise, and the exercise bout is such that Hb and Hct change in the following way:

**Table:** 

Hb_pre_ (g/L)	Hb_post_ (g/L)	Hct_pre_ (%)	Hct_post_ (%)
149	170	44	49

Assume further that the acute change from the exercise bout in the white cell count has been BM_pre_ = 4 × 10^9^/L →  5 × 10^9^/L = BM_post,*u*_ (+25%). Now, by applying the equation (eq. 1) to calculate the percentage of plasma shift, one gets ΔPV = −20.2%, and hence applying equation (eq. 3) a (wrongly) corrected value

$$\left(1+\Delta \text{PV}\right)\times {\text{BM}}_{\mathrm{post},u}=0.798\times 5\times 10^{9}/L=3.99\times 10^{9}/L.$$

Thus, using this “corrected” value of 3.99 × 10^9^/L based on plasma change, and comparing it to the initial value of 4 × 10^9^/L, one would deduce erroneously that ΔTBM ≈ 0, and that the plasma shift explains the whole observed change in the white cell count. However, by utilizing the correct equation (eq. 5) for the whole blood markers the true change is reached:

$$\Delta \text{TBM}=\frac{{\text{BM}}_{\mathrm{post}}}{{\text{BM}}_{\mathrm{pre}}}\times \frac{Hb_{\mathrm{pre}}}{Hb_{\mathrm{post}}}-1=\frac{5}{4}\times \frac{149}{170}-1=0.096=+9.6\%,$$

showing that the total count of circulating white cells in a bloodstream has, in fact, risen nearly 10% underlining the importance of choosing the right formula.

To explicitly illustrate that this is the right way, one can assume further that we know the blood volume BV_pre_ = 6 L. From this, applying (eq 2), it can be calculated that BV_post_ = 5.26 L. Hence the total count of circulating white cells from TBM = BM × BV are TBM_pre_ = 24 × 10^9^ and TBM_post_ = 26.3 × 10^9^ from which the total increase of +9.6% ($=\frac{26.3-24}{24}$) can be directly verified.

## Conclusion

The usually applied equation by Dill and Costill (eq. 3) is appropriate for calculating the corrections for plasma and serum biomarkers due to the change in plasma volume. However, it is not suitable for whole blood biomarkers, for which an alternative equation (eq. 5) should be preferred.

## Conflict of Interest

None declared.
